# Initial Phylotranscriptomic Confirmation of Homoplastic Evolution of the Conspicuous Coloration and Bufoniform Morphology of Pumpkin-Toadlets in the Genus *Brachycephalus*

**DOI:** 10.3390/toxins13110816

**Published:** 2021-11-19

**Authors:** Mariana L. Lyra, Juliane P. C. Monteiro, Loïs Rancilhac, Iker Irisarri, Sven Künzel, Eugenia Sanchez, Thais H. Condez, Omar Rojas-Padilla, Mirco Solé, Luís Felipe Toledo, Célio F. B. Haddad, Miguel Vences

**Affiliations:** 1 Instituto de Biociências, Departamento de Biodiversidade (Campus Rio Claro), Universidade Estadual Paulista (UNESP), Avenida 24A, N 1515, Bela Vista, Rio Claro 13506-900, SP, Brazil; marillyra@gmail.com (M.L.L.); julianepmonteiro@gmail.com (J.P.C.M.); haddad1000@gmail.com (C.F.B.H.); 2 Zoological Institute, Technische Universität Braunschweig, 38106 Braunschweig, Germany; loisrancilhac@gmail.com (L.R.); eu.sanisa@gmail.com (E.S.); 3 Institute for Microbiology and Genetics, Department of Applied Bioinformatics, University of Goettingen, Goldschmidtstr, 1, 37077 Göttingen, Germany; irisarri.iker@gmail.com; 4 Max Planck Institute for Evolutionary Biology, 24306 Plön, Germany; kuenzel@evolbio.mpg.de; 5 Unidade Passos, Universidade do Estado de Minas Gerais (UEMG), Avenida Juca Stockler 1130, Passos 37900-106, MG, Brazil; thacondez@gmail.com; 6 Laboratório de Sistemática de Vertebrados, Pontifícia Universidade Católica do Rio Grande do Sul, Av. Ipiranga 6681, Porto Alegre 90619-900, RS, Brazil; projasomar@gmail.com; 7 Departamento de Ciências Biológicas, Universidade Estadual de Santa Cruz, Ilhéus 45662-900, BA, Brazil; mksole@uesc.br; 8 Laboratório de História Natural de Anfíbios Brasileiros (LaHNAB), Departamento de Biologia Animal, Instituto de Biologia, Universidade Estadual de Campinas, Campinas 13083-862, SP, Brazil; toledosapo@gmail.com

**Keywords:** Amphibia, Anura, RNAseq, phylogenomics, mitochondrial genomes, mitochondrial gene order, convergence, aposematism

## Abstract

The genus *Brachycephalus* is a fascinating group of miniaturized anurans from the Brazilian Atlantic Forest, comprising the conspicuous, brightly colored pumpkin-toadlets and the cryptic flea-toads. Pumpkin-toadlets are known to contain tetrodotoxins and therefore, their bright colors may perform an aposematic function. Previous studies based on a limited number of mitochondrial and nuclear-encoded markers supported the existence of two clades containing species of pumpkin-toadlet phenotype, but deep nodes remained largely unresolved or conflicting between data sets. We use new RNAseq data of 17 individuals from nine *Brachycephalus* species to infer their evolutionary relationships from a phylogenomic perspective. Analyses of almost 5300 nuclear-encoded ortholog protein-coding genes and full mitochondrial genomes confirmed the existence of two separate pumpkin-toadlet clades, suggesting the convergent evolution (or multiple reversals) of the bufoniform morphology, conspicuous coloration, and probably toxicity. In addition, the study of the mitochondrial gene order revealed that three species (*B*. *hermogenesi*, *B*. *pitanga*, and *B. rotenbergae*) display translocations of different tRNAs (NCY and CYA) from the WANCY tRNA cluster to a position between the genes ATP6 and COIII, showing a new mitochondrial gene order arrangement for vertebrates. The newly clarified phylogeny suggests that *Brachycephalus* has the potential to become a promising model taxon to understand the evolution of coloration, body plan and toxicity. Given that toxicity information is available for only few species of *Brachycephalus*, without data for any flea-toad species, we also emphasize the need for a wider screening of toxicity across species, together with more in-depth functional and ecological study of their phenotypes.

## 1. Introduction

The evolution of aposematism, i.e the co-occurrence of conspicuous coloration and defensive mechanisms, is fascinating and poorly understood, despite intensive research over many decades (e.g., [1,2,3,4,5]). Whether an apparently bright and conspicuous colour pattern is aposematic (i.e., it has primarily a warning function) rather than serving intraspecific communication or even camouflage [1,6,7] and how warning colour integrates with the evolution of other phenotypic characters [8] are intriguing questions that can only be answered by subjecting a variety of animal systems to experimental (e.g., [9,10,11], phylogenetic (e.g., [12]), and genomic research (e.g., [13]).

**Figure 1:**
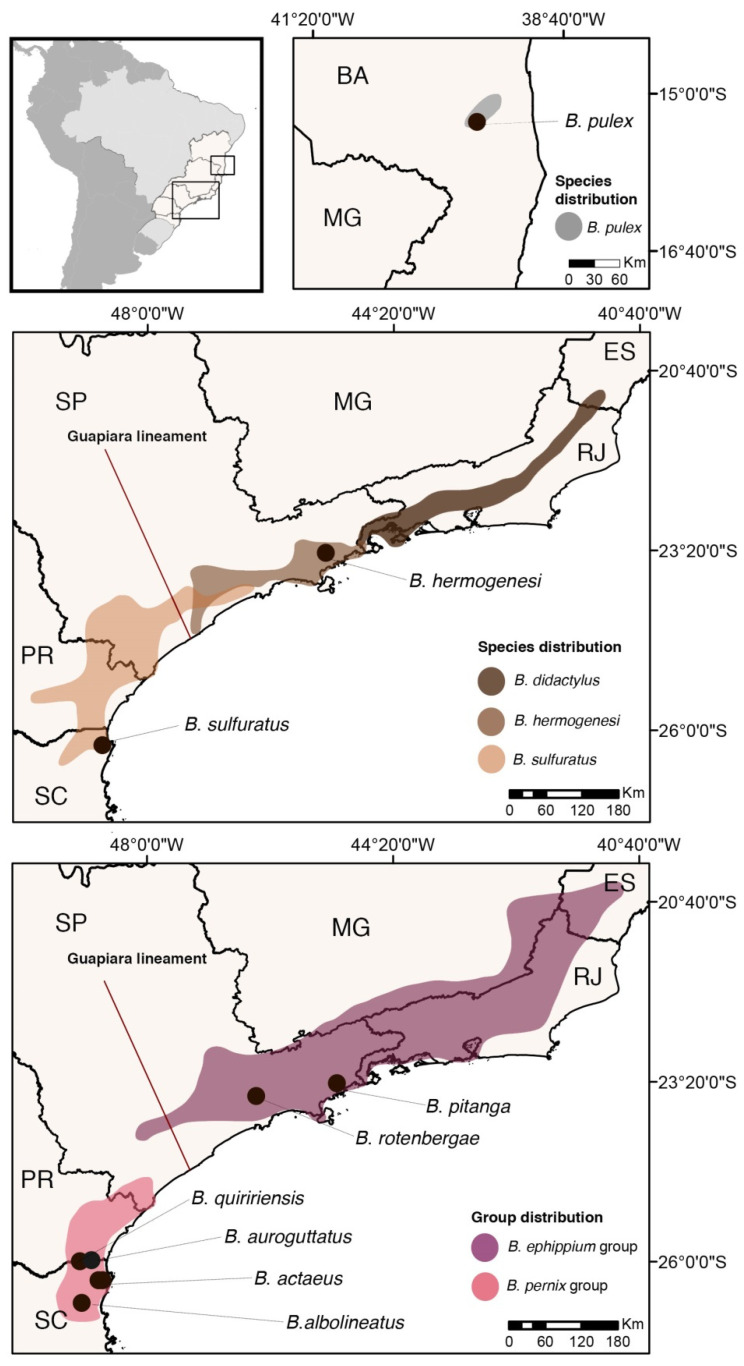
Approximate distribution map for genus *Brachycephalus* based on literature records, with sampling localities for each species included in the phylogenomic analysis. Acronyms for Brazilian states: BA, Bahia; ES, Espírito Santo; MG, Minas Gerais; PR, Paraná; RJ, Rio de Janeiro; SC, Santa Catarina; and SP, São Paulo.

Amphibians have long been a prime group for research on the evolution of aposematism and contain a large number of species that exhibit putative warning signals [14]. They are also the only class of terrestrial vertebrates known to contain tetrodotoxin analogues (TTX; [15]). One example is the Brazilian genus *Brachycephalus* which currently comprises 38 described species [16], all endemic to the Atlantic Forest biome between 15° and 26° latitude from sea level up to 2000 m [17] (Figure 1). Species in this genus are essentially diurnal, leaf-litter inhabitants, miniaturized, and characterized by direct development [18,19]. *Brachycephalus* species are divided into two groups according to their body plans, popularly known as pumpkin-toadlets (34 species) and flea-toads (4 species) [20]. All pumpkin-toadlets are characterized by a “bufoniform” appearance, with a robust body and pectoral girdle, head as wide as long and short snout; the flea-toads instead have a “leptodactyliform” appearance with slender body and pectoral girdle, head longer than wide and long snout (Supplementary Figure S1). The relatively more elongated skull of the flea-toads is also reflected in a skeletal multivariate morphospace [20]. The coloration of pumpkin-toadlets is usually bright, orange/yellow but sometimes greenish/brownish dorsally, and always with some orange components at least on the hands and feet and/or on the ventral surface. Several pumpkin-toadlets are also known to contain toxins: TTX has been reported in *B. ephippium*, *B. nodoterga*, *B. pernix*, *B. pitanga*, and *B. rotenbergae* (as *B. ephippium*) [15,21,22] and bradykinin-potentiating peptides (BPP) have been reported in *B. rotenbergae* ([23]—as *B. ephippium*). On the contrary, flea-toads have an inconspicuous brown coloration [19,20] and their putative toxicity has so far not been assessed. These characteristics make *Brachycephalus* an excellent model for studying the evolution of anti-predator defense mechanisms associated with toxins.

**Figure 2:**
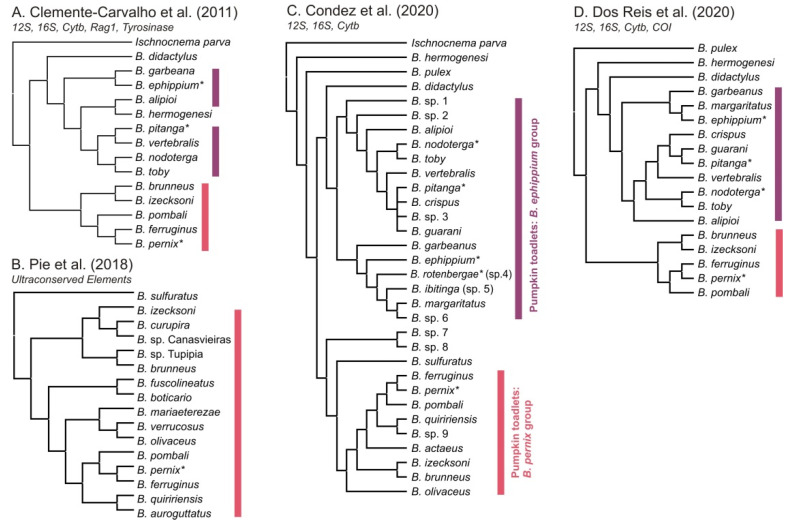
Summary of selected results from previous phylogenetic analyses of the genus *Brachycephalus*: (**A**) Bayesian inference (BI) analysis of a concatenated matrix 4563 bp of three mitochondrial and two nuclear gene fragments [18]; (**B**) BI analysis of 155,683 bp of 303 concatenated ultraconserved (UCE) loci [28]; (**C**) BI analysis of 2372 bp of three concatenated mitochondrial gene fragments [19] (**D**) species tree estimation from 4826 bp of four mitochondrial gene fragments [20]. Purple bars refer to the *B. ephippium* group and pink bars refers to the *B. pernix* group. Asterisks (*) mark species known to contain toxins (TTX). The two groups of pumpkin-toadlets are marked with vertical bars; other species are flea-toads (or outgroup: *Ischnocnema*). Note that *B. rotenbergae* has only recently been recognized as a distinct species and some sequences as well as toxin data assigned to *B. ephippium* in earlier studies may refer to this species.

Surprisingly, early molecular phylogenetic evidence (Figure 2A) suggested that these two morphological groups are not reciprocally monophyletic within *Brachycephalus* [18]. Based on DNA sequences of few mitochondrial and two nuclear-encoded genes (tyrosinase and recombination activating gene 1), two major clades of pumpkin-toadlets were recovered: the *B*. *ephippium* group (with 15 species), characterized by a gradient of increased mineralization in the skull [20,24,25,26,27], and the *B*. *pernix* group (with 19 species), which is not hyperossified [18,19,20]. The *B*. *ephippium* group is distributed across the Serra do Mar and Serra da Mantiqueira mountain ranges whereas the *B. pernix* group is distributed across the Serra do Mar, respectively at the north and south of the Guapiara Lineament [19] (Figure 1). However, the relationships of the flea-toad species within *Brachycephalus* (*B*. *didactylus*, *B*. *hermogenesi*, *B. pulex*, and *B*. *sulfuratus*) remain incompletely resolved (Figure 2). Mitochondrial analyses recovered *B. didactylus* sister to the *B*. *ephippium* group with high support, as well as *B*. *sulfuratus* sister to the *B*. *pernix* group [19,20]. *Brachycephalus hermogenesi* was recovered either nested within the *B*. *ephippium* group [18] or sister to a clade encompassing all other *Brachycephalus* with low support [19]. The northernmost distributed species *B*. *pulex* (Figure 1) seems to split from a deep node in the *Brachycephalus* tree [20], however, the relationship between this species and other *Brachycephalus* spp. are not well supported [19,20].

Although current knowledge suggests homoplasy of the aposematic coloration and body plan in pumpkin-toadlets, the conflicting and often poorly supported phylogenetic placements of various flea-toad species do not allow understanding whether the multiple instances of the pumpkin-toadlet phenotype represents homoplasy or shared ancestry. Here, we aim to assess the relationships within *Brachycephalus* with new phylogenomic data derived from RNAseq from representatives of each major clade of pumpkin-toadlets and three species of flea-toads, thus representing most of the main clades within the genus. We analyzed complete mitochondrial genomes reconstructed from the RNAseq data as well as a comprehensive phylotranscriptomic data set of over 5000 nuclear-encoded protein-coding genes, which resulted in largely congruent and supported hypotheses for the evolutionary history of *Brachycephalus.*

## 2. Results

### 2.1. Phylogenetic Relationships

**Figure 3:**
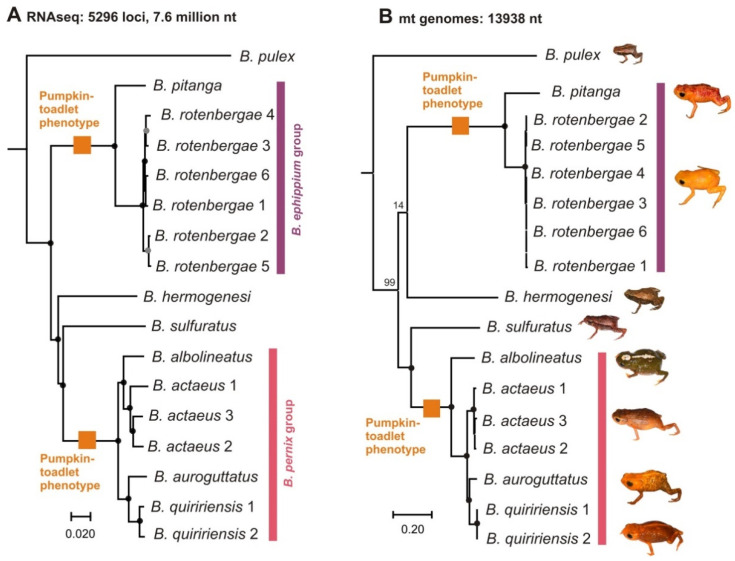
Phylogenomic trees of *Brachycephalus* spp. (**A**) Maximum Likelihood (ML) tree calculated from a concatenated alignment of 7,612,827 nucleotide positions (nt) of 5296 nuclear-encoded protein-coding markers derived from transcriptomes. Black circles at nodes indicate full support from both the analysis of the concatenated dataset (with IQTREE: 100% for 1000 aLRT pseudoreplicates) and from a species tree analysis (with ASTRAL: 1.0 local posterior probability); grey nodes received 100% support in the IQTREE analysis but a posterior probability <1.0 in the ASTRAL analysis. (**B**) Maximum likelihood tree calculated from a concatenated alignment of 13,938 nucleotide positions of the two rRNA genes and the 13 protein coding genes from full mitochondrial genomes. Black circles at nodes indicate 100% bootstrap support, other bootstrap values are written out (except for the most shallow intraspecific nodes). Orange squares mark clades composed of species characterized by the pumpkin-toadlet phenotype (bufoniform morphology, conspicuous orange coloration at least on some parts of the body, and probable toxicity), as opposed to the flea-toad phenotype (leptodactyliform morphology, cryptic coloration and possibly reduced toxicity) of *B. pulex, B. hermogenesi* and *B. sulfuratus.* Both trees were rooted with *Ischnocnema henselii* as outgroup (removed from the figure for better graphical representation of the trees). Scale bars represent expected substitutions per site.

The final phylotranscriptomic dataset consisted of 5296 alignments of ortholog nuclear-encoded protein-coding genes, totaling 7,612,827 aligned nucleotide positions. Partitioned ML analysis of the concatenated data set resulted in a fully resolved tree (Figure 3A) with full support on all internal branches, except for two intraspecific nodes connecting individuals of *B*. *rotenbergae*. The ASTRAL tree recovered the same topology with fully supported inter-specific relationships as well (Supplementary Figure S2). The ML analysis of the concatenated alignment of 13,938 mitochondrial nucleotide positions (the two rRNA and the 13 protein-coding genes) yielded a tree overall concordant to that obtained from the nuclear-encoded data (Figure 3B; Supplementary Figure S3), the disagreements affecting deep nodes that were poorly resolved by the mitogenomic data and the position of one species within the *B. pernix* group (*B*. *albolineatus*, see below).

The two data sets are concordant regarding the phylogenetic relationships of the flea-toads *B*. *pulex* and *B*. *sulfuratus*. *Brachycephalus pulex* is placed sister to all other included *Brachycephalus* with full support (100% aLRT in the nuclear tree, 99% bootstrap support in the mitochondrial tree; Figure 3). *Brachycephalus sulfuratus* is placed with maximum support in all analyses as sister to one clade of pumpkin-toadlets (the *B. pernix* group). The analyses also recovered the included species of the *B*. *ephippium* group, and of the *B*. *pernix* group, as monophyletic, respectively. The phylogenetic position of the flea-toad *B*. *hermogenesi* was contradictory. Whereas the phylotranscriptomic analyses recovered it sister to the clade containing the *B*. *pernix* group and *B*. *sulfuratus* with maximum support, the mitogenomic analysis placed it as sister to the *B*. *ephippium* group but without support (bootstrap proportion = 14%). The position of *B*. *albolineatus* within the *B*. *pernix* group was also different between the datasets: phylotranscriptomic analyses recovered the species as sister to *B*. *actaeus*, whereas the mitogenomic analysis placed it sister to the *B*. *pernix* group. These alternative topologies received maximum support by the analyses of the respective data sets. All species covered by multiple samples were monophyletic with high support and displayed short intra-specific branches.

### 2.2. Reconstruction of Phenotype Evolution

**Figure 4:**
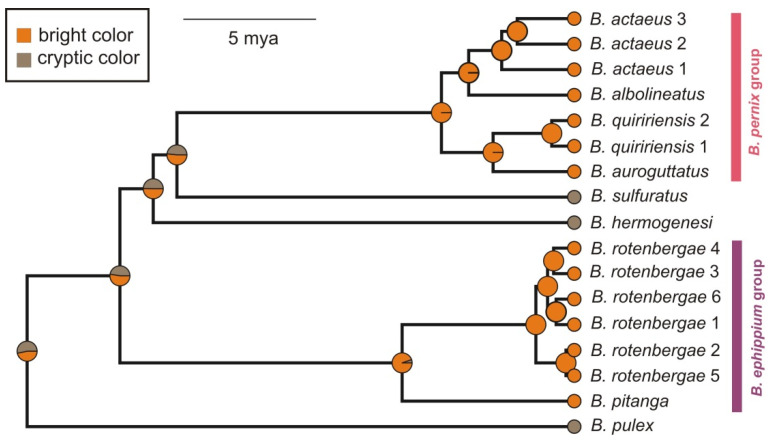
*Brachycephalus* phenotype reconstructed under a Bayesian (stochastic character mapping; SIMMAP) using phytools. Identical reconstructions were obtained for color (shown), and for bufoniform vs. leptodactyliform morphology. The character state “bright color” defines species that have at least some part of the body surface colored bright orange (dorsum, hands and feet, or ventral surface). Scale bar represents 5 million years it is based on calibration of the *Ischnocnema*/*Brachycephalus* split at 35 million years ago (see methods).

Reconstruction of ancestral character states via Bayesian stochastic character mapping yielded ambiguous results where most deep nodes in the tree had similar probabilities for both phenotypes (Figure 4), with a tendency of higher probabilities for bright color and bufoniform body plan. Similar results were found when the outgroup (*Ischnocnema*) was either included or excluded. An explorative parsimony analysis (Fitch optimization in TNT; [29]) reconstructed a convergent evolution of the pumpkin-toadlet phenotype, i.e., according to this approach, both the bufoniform body plan and bright coloration originated independently in the *B. ephippium* and the *B. pernix* group (Supplementary Figure S4).

### 2.3. Mitogenome Gene Order

**Figure 5:**
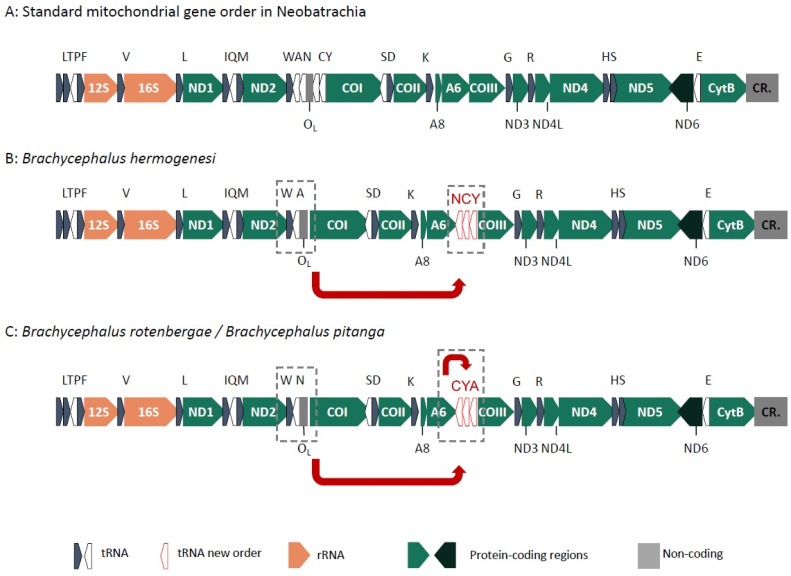
Schematic representation of mitochondrial DNA gene arrangement in *Brachycephalus*. Arrows represent rearrangements in relation to standard gene order of Neobatrachia. The black squares highlight the rearrangement region. Gene alias follows HUGO gene nomenclature committee (https://www.genenames.org/; accessed on 1 September 2021). (**A**) Standard mitochondrial gene order as observed in most Neobatrachia; (**B**) gene order observed in *B. hermogenesi*; (**C**) gene order observed in *B. rotenbergae* and *B. pitanga*.

Near-complete mitogenomes were recovered for all specimens, including the two ribosomal genes (12S and 16S rRNA), the 13 protein-coding genes, and most tRNAs (Figure 5). We also recovered partial control region (CR) sequences for *B. pitanga* and *I*. *henselii*. Most genes are transcribed from the H-strand, except for ND6 and eight tRNA genes, as known for other anurans [30,31]. We found only a few regions with low coverage in all mitogenomes, mostly representing partial tRNA regions and the 3′ or 5′ regions of coding-sequences. This pattern is expected when by-catching mitogenomes from RNAseq data.

The gene arrangement in *B. pulex*, *B. sulfuratus* and species of the *B. pernix* group follows the most common order known for Neobatrachia [31,32] (Figure 5A). However, two novel tRNA gene rearrangements were found for *B*. *hermogenesi* and for *B*. *pitanga* + *B*. *rotenbergae*. In these three species genes from the WANCY tRNA cluster are rearranged to the region between ATP6 and COIII. In *B*. *hermogenesi* the tRNAs N, C, and Y are translocated, probably in a single translocation event maintaining the same order found in the WANCY cluster (Figure 5B). In *B*. *pitanga* and *B*. *rotenbergae,* instead, the tRNAs A, C, and Y were found in between ATP6 and COIII, and furthermore the tRNA-A was found after tRNAs C and Y (Figure 5C). These new arrangements did not affect the position of the origin of replication for the light-strand synthesis (O_L_) (Figure 5). Strikingly, the other species of *Brachycephalus* also have a non-coding region between ATP6 and COIII, which is missing in the outgroup (*Ischnocnema*). Given the high coverage and quality of the fragment spanning between ATP6 and COIII in most assemblies, these arrangements are unlikely to be artefactual. In addition, the de novo assembly using the program mitoZ also recovered these arrangements, and PCR tests confirmed different length fragments for species of rearranged gene order vs. standard gene order (Supplementary Figure S5). 

## 3. Discussion

### 3.1. Evolution and Biogeography of Brachycephalus

The phylotranscriptomic and mitogenomic analyses conducted herein support that flea-toads do not represent a monophyletic group exclusive of pumpkin-toadlets. The analyses recovered *B. pulex* as the sister species to all other *Brachycephalus* with high support, as previously suggested [20]. The position of *B*. *sulfuratus,* sister to the *B. pernix* group, also agrees with previous studies based on mitochondrial DNA markers [19,20]. The phylogenetic position of *B*. *hermogenesi* was not unambigously solved by the analyses herein; the phylotranscriptomic dataset suggests a novel phylogenetic relationship of this species not recovered by previous studies, being the sister clade of *B*. *sulfuratus* + *B*. *pernix* group, whereas its position remained poorly supported in the mitogenomic tree. A single species of flea-toad, *B. didactylus*, is missing from our phylogenomic analyses. However, in all previous works, this taxon was consistently recovered with high support as sister to the *B. ephippium* group [18,19,20] and this position was also recovered in an analysis including these data along with our mitogenomic sequences (Supplementary Figure S6). Therefore, we expect this position will also be confirmed once phylogenomic data for this species become available.

Our results corroborate previous analyses in recovering two fully supported clades of conspicuous pumpkin-toadlets: (i) the *B*. *ephippium* group (represented by *B*. *pitanga* and *B*. *rotenbergae*), and (ii) the *B*. *pernix* group (represented by *B*. *actaeus*, *B. albolineatus*, *B. auroguttatus*, and *B. quiririensis*) [18,19,20]. As far as known, in *Brachycephalus* the pumpkin-toadlet phenotype includes two components that in all known species are linked (bufoniform morphology and conspicuous coloration), i.e., all bufoniform species of the genus are also more or less brightly colored (sometimes including green and brownish morphs). Stochastic character mapping could not reliably reconstruct the ancestral phenotype of *Brachycephalus* and the polarization of character states therefore remains ambiguous (Figure 4). The pumpkin-toadlet phenotype can be hypothesized to be derived given its absence in *Ischnocnema*, the sister clade of *Brachycephalus*, and along with other studies [20] we consider the hypothesis of convergent origin of this derived phenotype more likely, rather than postulating multiple reversals. We anticipate that this hypothesis will receive stronger support if the probable sister-group relationship of the *B. ephippium* clade and the flea-toadlet *B. didactylus* [19,20] (Supplementary Figure S6 herein) is confirmed by future phylogenomic data sets and included in the ancestral character state reconstruction, but at present it is clear that the details of the phenotype homoplasy in *Brachycephalus* cannot be resolved with full reliability.

Furthermore, the two pumpkin-toadlet clades show different patterns of ossification. In the *B. ephippium* group, species have remarkably ossified skulls and vertebral columns, sometimes with a bony dorsal shield, and bone sculpturing consisting of ridges and crests inducing a reticulated or pitted pattern in the skull, spinal processes of sacral, and presacral vertebrae [20,25]. In contrast, in the *B. pernix* group, such hyper-ossification is not observed [19,20,27]. This might indicate that the bufoniform appearance of these toadlets is ontogenetically based on different developmental processes, which would be in agreement with an independent evolutionary origin.

From a biogeographical perspective, a previous study [19] hypothesized that the ancestral distribution of *Brachycephalus* was in the northern part of its current range, specifically in high-elevation regions in the northern Serra do Mar mountain range. From the results obtained herein, we can refine this hypothesis relying on the assumptions that (i) our phylotranscriptomic tree correctly represents the evolutionary history of the main *Brachycephalus* clades, and (ii) the phylogenetic position of the flea-toad species *B. didactylus* has been correctly recovered by previous studies (see also Supplementary Figure S6). The flea-toad *B. pulex* was recovered sister to all other *Brachycephalus* spp. and occupies the northernmost distribution range (Figure 1). Hence, it is possible that the initial split in the genus occurred between a northern and central/southern ancestral Atlantic Forest species, both likely possessing a flea-toad morphology. In this hypothesis, the subsequent split may have separated two main further clades characterized by an ancestral flea-toad phenotype. One of these occupied much of the central part of the Atlantic Forest, with a widespread species of flea-toad morphology (*B. didactylus*) and one clade, the *B. ephippium* group, evolving the pumpkin-toadlet phenotype (Figure 1). The second clade would have dispersed southwards where successive stages of this colonization are represented by the flea-toad lineages, *B*. *hermogenesi* and *B*. *sulfuratus*, and the *B. pernix* group that evolved the pumpkin-toadlet phenotype (Figure 1).

### 3.2. Mitogenome Evolution and Cyto-Nuclear Discordance

Gene rearrangements comparable to those observed in *Brachycephalus* have been reported in all lissamphibian orders [30,33,34,35]. Especially the WANCY genomic region has been previously considered a hotspot for gene order rearrangements [33,34]. For example, the caecilians *Luetkenotyphlus brasiliensis* and some *Siphonops* spp. have different arrangements for tRNAs in this region (NCYWA and ACWNY, respectively) [34]. However, the partial rearrangement of some of these tRNA genes to the position between the ATP6 and COIII genes, as observed in several *Brachycephalus*, is new for vertebrates.

The mitochondrial gene order could be interpreted as supporting a close relationship of *B*. *hermogenesi* and the *B*. *ephippium* group, given that in both, genes from the WANCY tRNA cluster were translocated to the region between ATP6 and COIII, an unprecedented position for such translocations among frogs. However, since different tRNAs were affected by this translocation in these taxa, and furthermore in a different order, it is unlikely that the observed pattern reflects a single, ancestral translocation event. It is more likely that a complex history of multiple translocations and maybe translocation reversals affected the mitochonodrial genomes of *Brachycephalus* species, as supported by the presence of non-coding DNA at this position in all species studied, including the earliest diverging lineage, *B. pulex*. It also cannot be fully ruled out that the shared position of the translocated tRNA clusters in *B*. *hermogenesi* and the *B*. *ephippium* group might be due to an early mitochondrial introgression event among ancestral *Brachycephalus* lineages.

The low support found for the deep nodes in the mitogenome tree suggests that mitochondrial data alone–even complete mitochondrial genomes–might be insufficient to reliably resolve several of the deepest nodes in the *Brachycephalus* tree. However, in one case, we detected a clear disagreement between phylotranscriptomic and mitogenomic trees: the mitogenomic tree placed *B*. *albolineatus* sister to all other included species of the group (in general agreement with the mitochondrial tree of [36]), whereas the phylotranscriptomic tree suggested a sister-group relationship to *B*. *actaeus*. This cytonuclear discordance in the placement of *B*. *albolineatus* might reflect inter-lineage gene flow. Our phylotranscriptomic tree includes only a small subset of species of the *B*. *pernix* group but according to the available mitochondrial gene trees (e.g., [36]) all species in the group are closely related, with maximum uncorrected pairwise distances in the 16S rRNA gene of <5.7%, and <1.4% between many closely related species [36] (Supplementary Figure S6). *Brachycephalus albolineatus* is closely related to *B*. *boticario*, *B*. *fuscolineatus*, and *B*. *mirissimus* based on mitochondrial data ([37]; present study), and a mitochondrial introgression from one of those species could explain the observed discordance. Alternatively, the species could have conserved a mitochondrial genome reflecting its evolutionary origins and its original nuclear genome diluted by frequent gene flow from other lineages close to *B*. *actaeus.* In amphibians, a similar situation of a divergent “ghost” mitochondrial genome not reflecting the evolutionary affinities of the nuclear genome was found for example in the salamander *Salamandra salamandra longirostris* from Spain [38,39]. Salamandrids have also served as a model to demonstrate pervasive hybridization among ancestral amphibian lineages [40], a phenomenon that might also affect the phylogeny of the genus *Brachycephalus*.

### 3.3. Challenges and Perspectives for Future Taxonomic and Evolutionary Studies

In our study, the intraspecific genetic variation found for the species with more than one sample analysed was very low. Particularly, for *Brachycephalus actaeus*, we included samples from different collecting points with remarkable colour variation (green, brownish, and orange, Supplementary Figure S1). Both datasets recovered *B*. *actaeus* as monophyletic with very short branches, providing further evidence for a polytypic species with substantial intra-specific colour variation. One of the phenotypes (orange) is more similar to that of other orange-colored species of the *B. pernix* group such as *B. mirissimus*. This example illustrates a new challenge to the taxonomy of this genus and may be caused by different populations of a species being subject to distinct selective pressures, translated into different frequencies of a dazzling variety of colour phenotypes. There are a few examples of similar systems in anurans, including poison-frogs of the families Dendrobatidae [12,13] and Mantellidae [41,42].

We flag as priorities for future investigation the inclusion of additional species such as *B. didactylus* in the phylotranscriptomic analysis, as well as including more specimens per species to better understand the limits of genetic and geographical variation within polytypic *Brachycephalus* species. It is also worth considering that several of the branches at the base of the phylogenomic tree are very short, including those that are most informative regarding the evolution of the pumpkin-toadlet phenotype, and these relationships may easily be influenced by introgression and thus be less stable than they appear in our tree. Once the crucial missing taxon (*B. didactylus*) has been included in the phylogenomic data set and its relationships reliably reconstructed, it will be crucial to perform introgression tests to better understand the origins of cyto-nuclear discordance and to provide a fully reliable test of the hypothesis of homoplastic evolution of this phenotype. For this purpose, a more in-depth characterization of the phenotypical groups is also needed. This should include objective assessments of color brightness, ideally with reflectance measurements against the background of the species’ habitats, as well as quantification of the body shape differences between pumpkin-toadlets and flea-toads.

While *Brachycephalus* have the potential to become a promising model taxon to understand the evolution of coloration, body plan and toxicity, comprehensive analyses are currently hampered by the poor taxonomic coverage of toxin analyses. Only few species of *Brachycephalus* have been screened for TTX and other toxic compounds, and most importantly, not a single flea-toad species has so far been included in these screens. It is therefore important to conduct a more extensive toxin screening, both in pumpkin-toadlets and flea-toads, to understand if bright colour in this genus is linked to toxicity and/or presence of TTX. In the same context, a broad experimental assessment of their supposed unpalatability to predators and the functions of their bony plates and associated fluorescent skeleton [11,43] are desirable to complement what we know about the complex evolutionary history of *Brachycephalus*.

## 4. Materials and Methods

### 4.1. Genetic Sampling 

Our sampling includes 18 individuals from nine species representing the main clades within *Brachycephalus* (*B*. *actaeus*, *B*. *albolineatus*, *B*. *auroguttatus*, *B*. *hermogenesi*, *B*. *pitanga*, *B*. *pulex*, *B. quiririensis*, *B*. *rotenbergae*, and *B*. *sulfuratus*; Table S1; Supplementary Figure S1) and one outgroup taxon (*Ischnocnema henselii*, a representative for the sister genus *Ischnocnema*; [44]; Supplementary Figure S1). Individuals were collected in the field, euthanized by applying 5% lidocaine to the skin, and the specimens were stored in RNAlater at −80 °C until RNA extraction. Specimens were collected under collection permits issued by ICMBio (Instituto Chico Mendes de Conservação da Biodiversidade; SISBio #13708-2, #17242-5, #49587-7, and #59889-1) and we registered the access to genetic information on the National System for the Management of Genetic Heritage and Associated Traditional Knowledge (SISGen #A58BC2D).

RNA was extracted from about 20–100 mg of tissue (combined or separate skin, muscle, or liver), and the extractions were performed using a standard TRIzol protocol as specified in the Supplementary Methods. Libraries for sequencing were prepared with the Illumina TruSeq Stranded mRNA Library Prep protocol (San Diego, CA, USA). The double-barcoded libraries were sequenced on an Illumina NextSeq instrument at the Max-Planck Institute for Evolutionary Biology in Plön, Germany, in multiple 150 bp or 75 bp paired-end runs (along with other samples of amphibians and reptiles not used for this study) each of which combined 10–14 samples per High-Output NextSeq kit. For species with transcriptomes of multiple individuals (*B*. *actaeus*, *B*. *quiririensis*, and *B*. *rotenbergae*) we chose to analyse each sample separately to assess the performance of our phylotranscriptomic approach in recovering species-level lineages. Reads were quality-trimmed and filtered using Trimmomatic v. 0.32 [45] with default settings, and transcriptomes were de novo assembled using Trinity v. 2.1.0 [46], following published protocols [47].

### 4.2. Phylotranscriptomic Analyses

To extract orthologous genes from the transcriptomes, we relied on the jawed-vertebrate alignment from [48] which included representative taxa of chondrichthyan and actinopterygian fishes, lungfish, coelacanth, amphibians, reptiles, birds, and mammals. This original data set consisted of a total of 100 taxa and was obtained by inferring putative orthologs from 21 reference proteomes representing most major clades of jawed vertebrates, and enriching them with genome and transcriptome data from 79 additional taxa using the software “42” (D. Baurain, https://metacpan.org/release/Bio-MUST-Apps-FortyTwo; accessed on 27 February 2021). The software “42” adds sequence data to the preexisting multiple sequence alignments (MSAs) after controlling for orthology using strict three-way reciprocal best BLAST hit tests, relying on a set of reference taxa available in the MSAs (*query_orgs*) and as complete proteomes (*ref_orgs*). The software performs a first BLAST search between *query_orgs* and *ref_orgs* and produces a database of best hits (*query_best_hits*). Then, a second BLAST search uses *query_orgs* to search the new transcriptomes to be added (*org*). Eventually, the identified homologs are BLAST-searched against *ref_orgs* and homologs are considered orthologs if the best hit with each of the reference proteomes is among the sequences in the *query_best_hit* list built earlier. The orthologs identified via this process are then added to the original MSAs, followed by multiple sequence alignment and redundancy filtering.

For the present study, we used the original 100-taxa dataset of [48] and enriched it with the newly sequenced *Brachycephalus* and *Ischnocnema* transcriptomes, using a new, separate round searches and alignments with the software “42”. After the protein-level alignment, we searched for sequences from the newly added data (*Brachycephalus* and *Ischnocnema*) that could represent putative contamination from prokaryotes and invertebrates added during the enrichment step. Such contaminant sequences might be added due to their conserved protein motifs or because they represent highly conserved genes throughout the tree of life. Contaminants were identified as significant BLAST hits against a custom protein database, and removed from the alignment.

Subsequently, for each locus the original nucleotide sequences were retrieved from the transcriptomes using leel (D. Baurain, https://metacpan.org/release/Bio-MUST-Apps-FortyTwo; accessed on 27 February 2021). Because data sets derived from low coverage transcriptomes or genomes may contain erroneous sequences caused by misassembly, frameshifts due to sequencing error, or recombination, and paralogs may also remain, a thorough decontamination procedure is necessary for which we employed tree-based orthology inference methods (e.g., [49]), as follows: (1) at the transcript level, highly fragmented sequences were removed (as very short transcripts are likely to produce phylogenetic artefacts simply because of too little information) and single-gene trees were inferred using RAxML v.8 [50] under a GTR + Γ substitution model. In-paralogs were identified based on long internal branches and removed, which represents a usual approach in phylogenomics. Sequences with very long terminal branches (in practice longer than the 99% quantile of the terminal branch length distribution) were interpreted as containing sequence errors or frameshifts and also removed. (2) Next, sequences were re-aligned with MAFFT [51], transcripts were merged with ScaFos v1.25 [52] and gene trees, as well as a concatenation tree, were newly inferred. (3) Individual taxa were removed from single-gene alignments when the exclusion significantly reduced the topological distance between the gene and concatenation trees (Robinson-Foulds distance). In this step, we set very stringent thresholds and manually checked every gene tree from which sequences were to be excluded and proceeded with the exclusion only when gene tree/concatenation tree differences could not be plausibly explained by biological processes such as incomplete lineage sorting or introgression. (4) Lastly, one additional cleaning step identical to step 2 was performed. From the obtained alignments, only those with five or more *Brachycephalus* samples were kept, and positions with >75% missing data were removed. Exploratory trees calculated before these decontamination steps agreed in all relevant nodes with our final tree, suggesting that these very conservative procedure has not biased our phylogenetic results.

A maximum likelihood (ML) tree was inferred from the concatenated alignment using IQ-TREE v1.6.8 [53,54], with best-fitting substitution models and gene partitions selected with BIC in ModelFinder [55], as implemented in IQ-TREE. Branch support was assessed with 1000 pseudoreplicates of SH-like approximate likelihood ratio test (aLRT). Furthermore, to account for possible effects of incomplete lineage sorting on phylogenetic inference, we also inferred a tree from the full data set using ASTRAL-II [56], a summary-tree method statistically consistent with the multispecies coalescent. Gene trees used as input for ASTRAL were inferred using RAxML under a GTR + Γ model. Branch support of the ASTRAL tree was assessed using local posterior probabilities.

### 4.3. Mitochondrial Genome Analyses 

The trimmed RNAseq reads were also used to recover mitochondrial DNA sequences. Paired-end FASTQ files were interleaved, and duplicates removed using Tally v. 14-020 [57]. We assembled mitochondrial genomes by iterative mapping using MITObim v. 1.9.1 [58], which internally uses MIRA v.4.0.2 [59]. The assembly was done in three steps: we first used the complete mitochondrial sequence of *Ischnocnema henselii* (GenBank accession number: MH492733) as initial seed and the mismatch parameter was set to 30 for all samples. Iterations were run until no additional reads could be incorporated into the assemblies. We evaluated the assemblies for completeness and quality by importing the mapping output from MITObim into Geneious R11 (https://www.geneious.com; accessed 1 February 2021). Since it was not possible to retrieve all coding genes for some *Brachycephalus* samples, we selected the most complete *Brachycephalus* mitogenome from the first round and used it as seed to reassemble all *Brachycephalus* samples, setting the mismatch parameter to 10. Finally, we used mitogenomes of each species as retrieved in the second round as seed for a third round of assembly to improve coverage and fill gaps. We did not use the published mitogenome of *Brachycephalus brunneus* as seed (GenBank accession KY355081) because the gene order of the available sequence does not match the gene order described in the original publication [60].

We also used mitoZ [61] to de novo assemble the mitogenome of some species and visually inspected them to confirm new gene arrangements (see results). We furthermore inspected read alignments visually to exclude assembly artefacts, and carried out some further tests to verify gene orders by designing primers spanning the tRNA WANCY region and performing PCRs tests to assess the length of the amplified fragment (Supplementary Methods).

Mitogenome annotations were performed in mitoZ [61] and the annotated contigs were loaded into Geneious R11 [62]. The two rRNAs (12S rRNA and 16S rRNA) and the 13 protein-coding genes were extracted, and the protein coding genes were checked to confirm that no indels or stop codons were present. Sequences of each gene were aligned with MAFFT [51] and aligned sequences were concatenated using SequenceMatrix v1.7.8 [63] for phylogenetic inference.

Data was partitioned by gene and by codon position when appropriate and the best partition scheme was selected using PartitionFinder 2 [64] (Table S2). A mitochondrial ML tree was inferred using RAxML v. 8.2.11 [50], with 100 ML searches and 1000 non-parametric bootstrap replicates, in the CIPRES Science Gateway (http://www.phylo.org; accessed 1 February 2021). The GTR + Γ substitution model was used for all partitions. Additionally we inferred mitochondrial trees using both ML and Bayesian Inference for all *Brachycephalus* species available from GenBank, to further explore hypotheses of phylogenetic relationships among flea-toads and pumpkin toadlets with maximum species coverage (Supplementary Methods; Supplementary Figure S4).

### 4.4. Reconstruction of Phenotype Evolution

Ancestral character states for body shape and colour were reconstructed using two approaches: a Bayesian Markov chain Monte Carlo approach to sample character histories from their posterior probability distribution (stochastic character mapping) [65]. For this we first transformed the phylotranscriptomic tree into an ultrametric tree using the program pyr8s within the iTaxoTools package [66], fixing the root node (*Brachycephalus/Ischnocnema* split) at an age of 35 million years ago based on a comprehensive anuran timetree [67]. We then used the function “make.simmap” from the R package phytools [68], performing 1000 simulations. Furthermore, we explored a parsimony-based [69] optimization as implemented in TNT v.1.5 [29], also based on the (untransformed) phylotranscriptomic tree.

For reconstruction of color phenotypes, we defined species with bright color as those that have at least some part of the body surface colored bright orange: i.e., even some pumpkin-toadlets with dorsal green or brownish color have orange on hands and feet, and/or part of the ventral surface, while orange color is not observed in any of the flea-toads. Body shape phenotypes were classified relying on previous classifications (e.g., [20]) of species as bufoniform (robust body and pectoral girdle, head as wide as long and short snout) vs. leptodactyliform (slender body and pectoral girdle, head longer than wide, and long snout).

## Data Availability

The raw RNAseq reads are available at the National Center for Biotechnology Information (NCBI) Sequence Read Archive (SRA) (BioProject ID number PRJNA742912). The mitochondrial sequences were submitted to GenBank (accession numbers MZ770735 -MZ770752; Table S1). All assemblies, alignments and tree files were uploaded to Figshare under the DOI 10.6084/m9.figshare.14884362.

## Supplementary Materials

The following are available online at https://www.mdpi.com/article/10.3390/toxins13110816/s1: Supplementary Methods (RNA extraction protocol, PCR tests including new primers design for WANCY region and mitochondrial DNA inference); Figure S1, Species sampled for this study in life. Figure S2, Species tree obtained using ASTRAL. Figure S3, Maximum likelihood tree calculated from mitogenomes. Figure S4, Maximum Likelihood mitochondrial tree for all available *Brachycephalus* species. Figure S5, Eletrophoresis gel showing different lengths of amplified fragments including the tRNA WANCY cluster. Figure S6, Maximum Likelihood phylogenetic inference for Brachycephalus species including mito-genomes obtained here and sequence data from GenBank. Table S1, List of samples used in the present analysis. Table S2. Best partition scheme selected for the mitogenome phylogenetic analysis. Table S3, Genbank accession numbers for species included in the mitochondrial ML and BI trees shown in Figure S4.
