# Supplementary material for: Initial Phylotranscriptomic Confirmation of Homoplastic Evolution of the Conspicuous Coloration and Bufoniform Morphology of Pumpkin-Toadlets in the Genus Brachycephalus
Source: Toxins (Basel). 2021 Nov 19;13(11):816. doi: 10.3390/toxins13110816 (PMC8620806; doi:10.3390/toxins13110816)
Supplement: Supplementary file 1 [file toxins-13-00816-s001.zip › toxins-1388060-supplementary.pdf]

toxins

MDPI

Supplementary Materials: Initial Phylotranscriptomic Confirmation of Homoplastic Evolution of the Conspicuous Coloration and Bufoniform Morphology of Pumpkin-Toadlets in the Genus *Brachycephalus*

Mariana L. Lyra, Juliane P. C. Monteiro, Loïs Rancilhac, Iker Irisarri, Sven Künzel, Eugenia Sanchez, Thais H. Condez, Omar Rojas-Padilla, Mirco Solé, Luís Felipe Toledo, Célio F. B. Haddad and Miguel Vences

Supplementary Methods

RNA extraction protocol:

Tissues (20–100 mg) from each sample were homogenized in 2 mL tubes with steel beads in 1 mL TRIzol using the tissue lyser Precellys (speed 6000, 2× 55 sec in between 10 sec break). The samples were centrifuged for 10 min at 12,000 g at 4 °C. The solution was transferred to a new tube and incubated at room temperature (RT) for 5 min, after which 200 µl chloroform was added, samples were vortexed for 15 sec, incubated for 3 min at RT and centrifuged for 15 min at 12,000 g at 4 °C. The upper phase was transferred to a new tube and 500 µL isopropanol was added. The solution was incubated for 10 min at RT and centrifuged for 15 min at 12,000 g at 4 °C. The supernatant was removed, 1 ml 75% EtOH was added to the pellet (using DEPC water) and the sample was vortexed until the pellet was resuspended. The samples were centrifuged for 5 min at 7500 g at 4 °C and the supernatant was removed. The pellet was dried and dissolved in DEPC water. The RNA was precipitated by adding 200 µL 5M LiCl solution and incubated for 1.5 h at −20 °C. After incubation, the solution was centrifuged for 20 min at 14,000 g at 4 °C. The supernatant was removed, the pellet washed with 500 µL 75% EtOH and then centrifuged for 10 min at 14,000 g at 4 °C. The pellet was again dried and dissolved in DEPC water.

Mitochondrial tRNA WANCY cluster amplification:

For verifying the gene order arrangement, independently of the *in silico* mitogenome DNA assembly algorithms, we performed amplification tests to assess the length variation of the WANCY region from different species. We first extracted total DNA from new individuals of the analyzed species using a standard ammonium acetate precipitation method [S1] (adapted by [S2]). Then, we designed two new primers flanking the WANCY region based on the sequences obtained from mitogenomes, one at the 3′ region of the ND2 gene (ND2_F2L_Brachy: 5′-ACCYTRGCCCCAAAY-TCAAC-3′) and one at the 5′ region of the COI gene (CO1_RH_Brachy: 5′-ARATTATYACGAAGGCGTG-3′). Finally, we PCR amplified the specimens using the following protocol: The amplification reactions were carried out in a 20-µL reaction, using the Ampliqon Taq DNA Polymerase Master Mix, 0.5 uL of each primer (10 µM) and 1 uL of extracted DNA. The thermal cycling conditions were: 3 min at 95 °C, 40 cycles of 20 s at 95 °C, 20 s at 50 °C, and 1min 20 s at 68 °C, and a final extension of 3 min at 68 °C.

The fragments were separated by electrophoresis in 1.5 % agarose gel, stained with GelRed (Bioyium inc.). The size of the fragments was estimated by comparison with the molecular size standard DNA Ladder GeneRuler 1 Kb (Thermo Scientific).

Mitochondrial phylogenetic inference for the genus Brachycephalus

To infer the mitochondrial phylogenetic relationship between species of the genus *Brachycephalus*, we used two optimality criteria: Bayesian inference (BI) and maximum likelihood (ML). For both analyses we gathered mitochondrial sequences (fragments of the 12S rRNA gene, 16S rRNA gene and Cytochrome B) from GenBank for one or two individuals per species available (Table S4) and extracted only these genes from the mitogenomes available We only used the mitochondrial sequences to avoid many missing data on data matrix. Final matrix contained 47 individuals and 3850 base-pairs.

Genes were aligned using MAFFT v7.130b [S3] and concatenated using SequenceMatrix [S4]. We computed BI analysis in MrBayes 3.2.6 [S5] using two independent runs of 10 × 106 generations, starting with random trees and eight Markov chains (one cold), sampled every 1000 generations. We performed the run using GTR+Γ model. We used the standard deviation of split frequencies (<0.01) and Estimated Sample Size (ESS > 200) to assess run convergence. We discarded 25% of generations and trees as burnin and obtained he 50% majority rule consensus tree. We computed ML analysis in RAxML v.8.2.10 [50], using GTR+Γ model, searching the most likely tree 1000 times and conducting 1000 non-parametric bootstrap replicates. We used *Ischnocnema henselii* as the root for all analyses, and we drew the trees using FigTree 1.4.2 [S6]. We performed all phylogenetic analyses using the CIPRES Science gateway portal [S7].

Supplementary Materials References

S1. Maniatis, T.; Fritsch, E.F.; Sambrook, J. *Molecular Cloning: A Laboratory Manual*. Cold Spring Harbor Laboratory Press, New York, 1982.

S2. Lyra, M.L.; Haddad, C.F.B.; de Azeredo-Spin, M.L. Toward defining the course of evolution: minimum change for a specific tree topology. *Mol. Ecol.* **2017**, *17*, 966–980, doi: 10.1111/1755-0998.12648.

S3. Katoh, K.; Standley, D.M. MAFFT multiple sequence alignment software version 7: improvements in performance and usability. *Mol. Biol. Evol.* **2013**, *30*, 772–780, doi:10.1093/molbev/mst010.

S4. Vaidya, G.; Lohman, D.J.; Meier, R. SequenceMatrix: concatenation software for the fast assembly of multigene datasets with character set and codon information. *Cladistics* **2011**, *27*, 171–180.

S5. Ronquist F.; Teslenko, M.; Van Der Mark, P.; Ayres, D.L.; Darling, A.; Hohna, S.; Larget, B.; Liu, L.; Suchard, M.A; Huelsenbeck, J.P. Mrbayes 3.2: efficient Bayesian phylogenetic inference and model choice across a large model space. *Syst. Biol.* **2012**, 61, 539–542, doi: 10.1093/sysbio/sys029.

S6. Rambaut, A.; Suchard, M.A.; Xie, D.; Drummond, A.J. Tracer v1.6. **2014**. University of Edinburgh, UK.

S7. Miller, M.A.; Pfeiffer, W.; Schwartz, T. Creating the CIPRES Science Gateway for inference of large phylogenetic trees. *Proc. Gatew. Comput. Environ. Workshop*, 2010, 1–8, doi: 10.1109/GCE.2010.5676129.

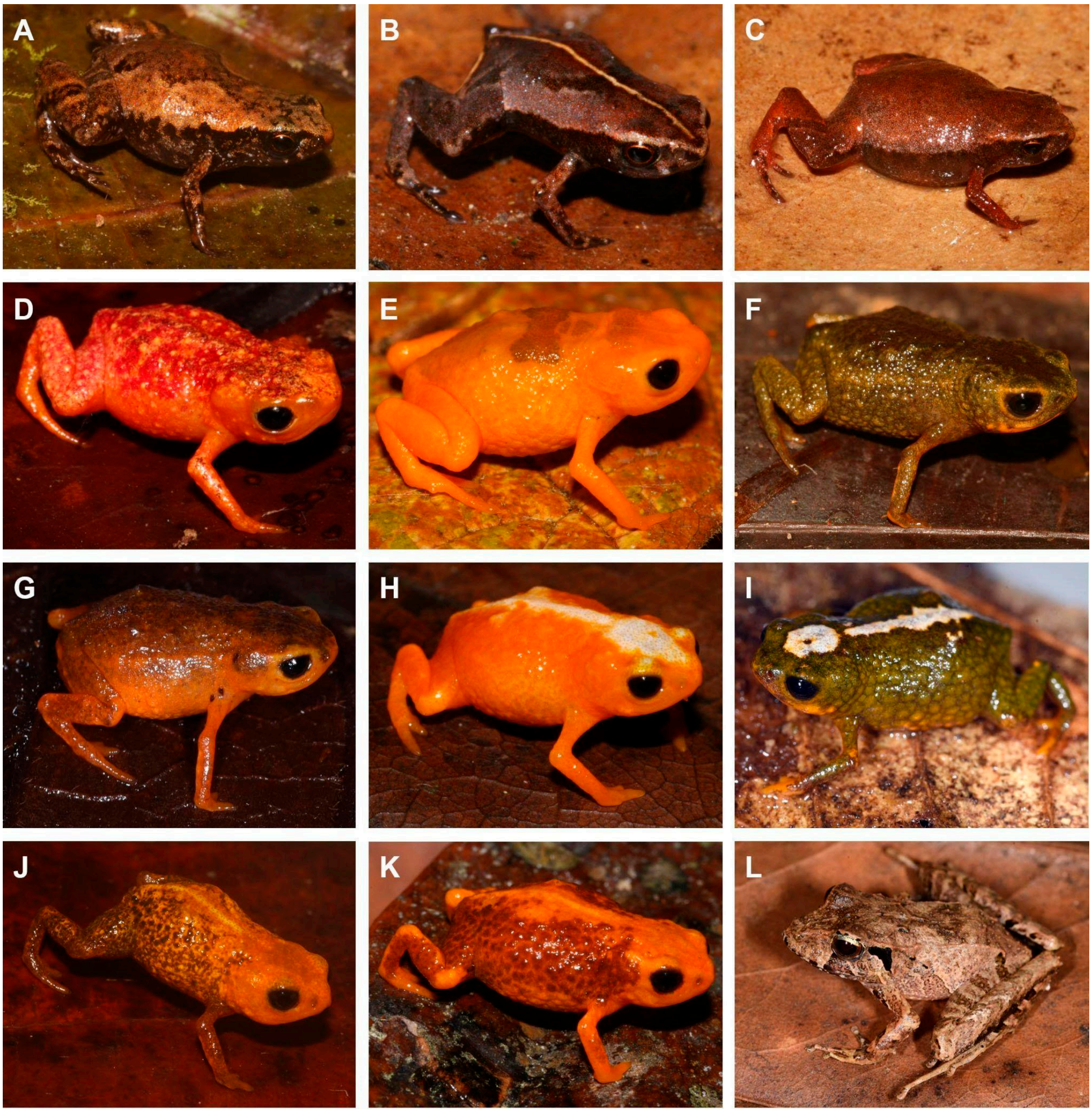

Figure S1. Species sampled for this study as representatives of the main clades within *Brachycephalus* (A–K) and species used as outgroup taxon, *Ischnocnema henselii* (L). The flea-toads with leptodactyliform body plan: (A) *B. hermogenesi*; (B) *B. pulex*; and (C) *B. sulfuratus*. The pumpkin-toadlets with bufoniform body plan of the *B. ephippium* group (D–E) and of the *B. pernix* group (F–K): (D) *B. pitanga*; (E) *B. rotenbergae*; (F–H) *B. actaeus*, morphotypes green, brownish, and orange, respectively; (I) *B. albolineatus*; (J) *B. auroguttatus*; and (K) *B. quiririensis*. Credits for the photos: Condez, TH (A–C, E, F, H, J, and K); Monteiro, JPC (D); and Vences, M (G, I, and L).

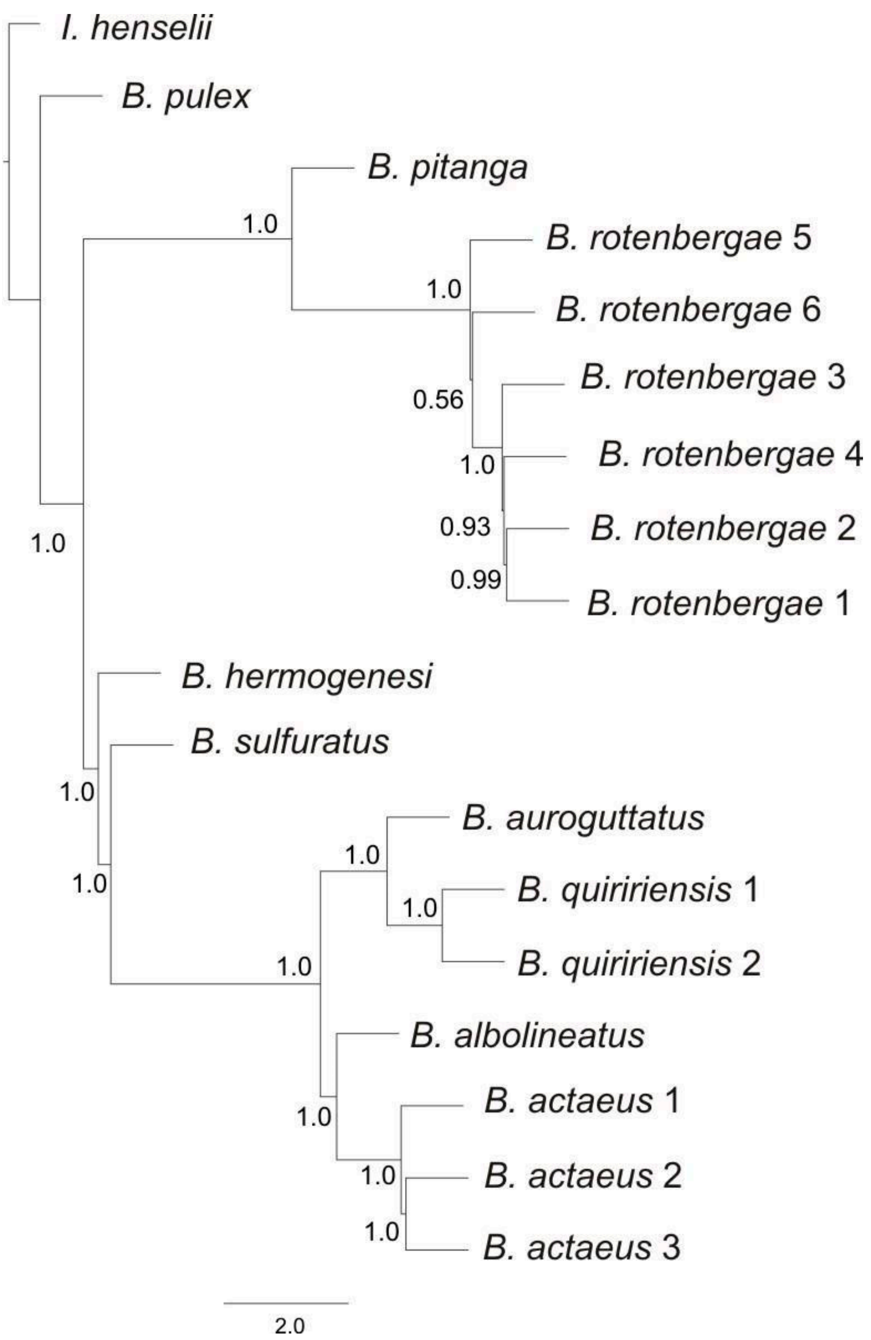

Figure S2. Species tree obtained using ASTRAL from 5296 gene trees of nuclear-encoded protein-coding markers derived from transcriptomes. Numbers at nodes show local posterior probabilities.

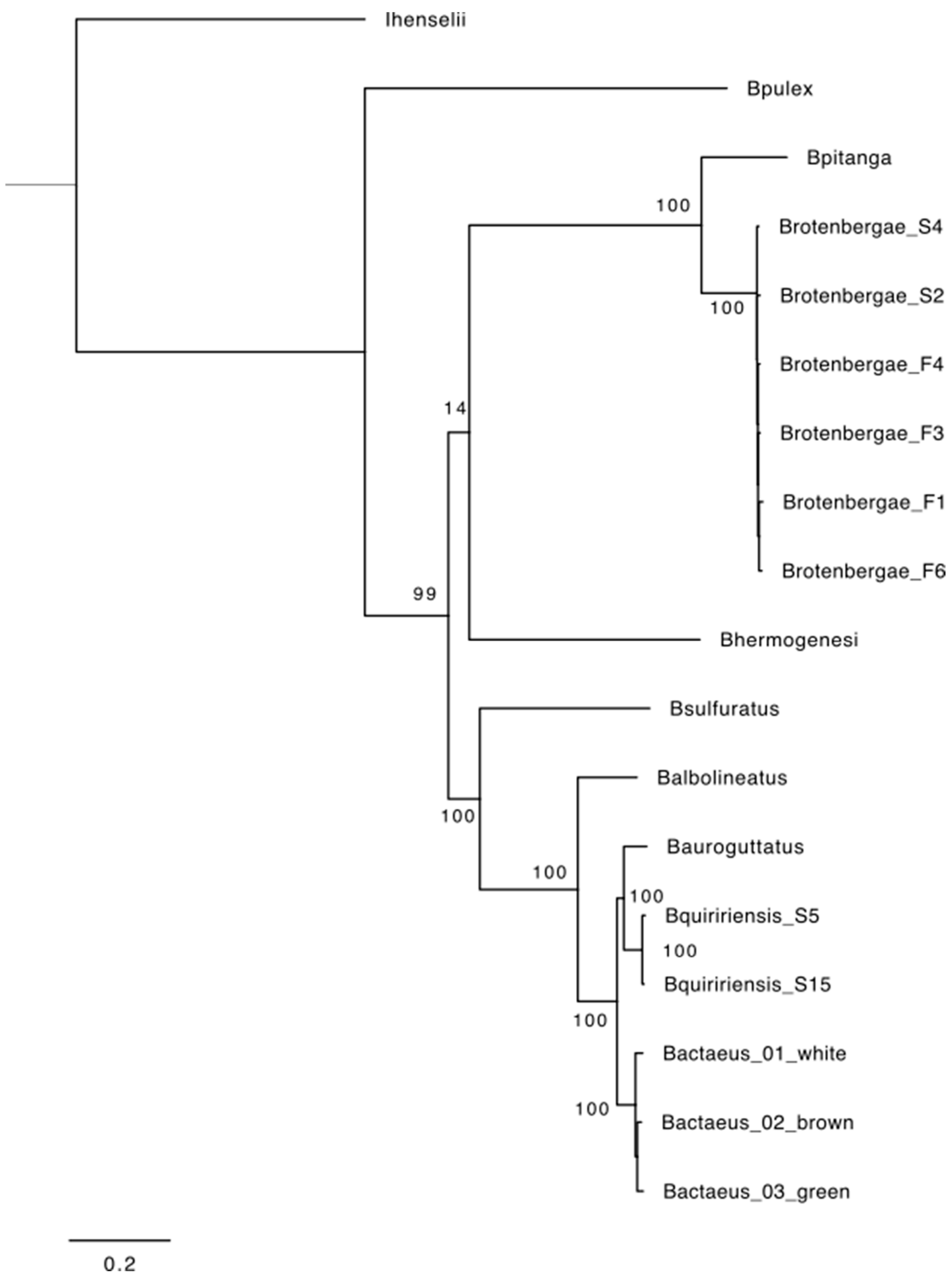

Figure S3. Maximum Likelihood tree calculated from a concatenated alignment of 13,938 nucleotide positions of the two rRNA genes and the 13 protein coding genes from full mitochondrial genomes. The tree represents a detailed version of Fig. 3A in the main paper, and is here shown to give full information on outgroup and node support values, as non-parametric bootstrap support shown at each node.

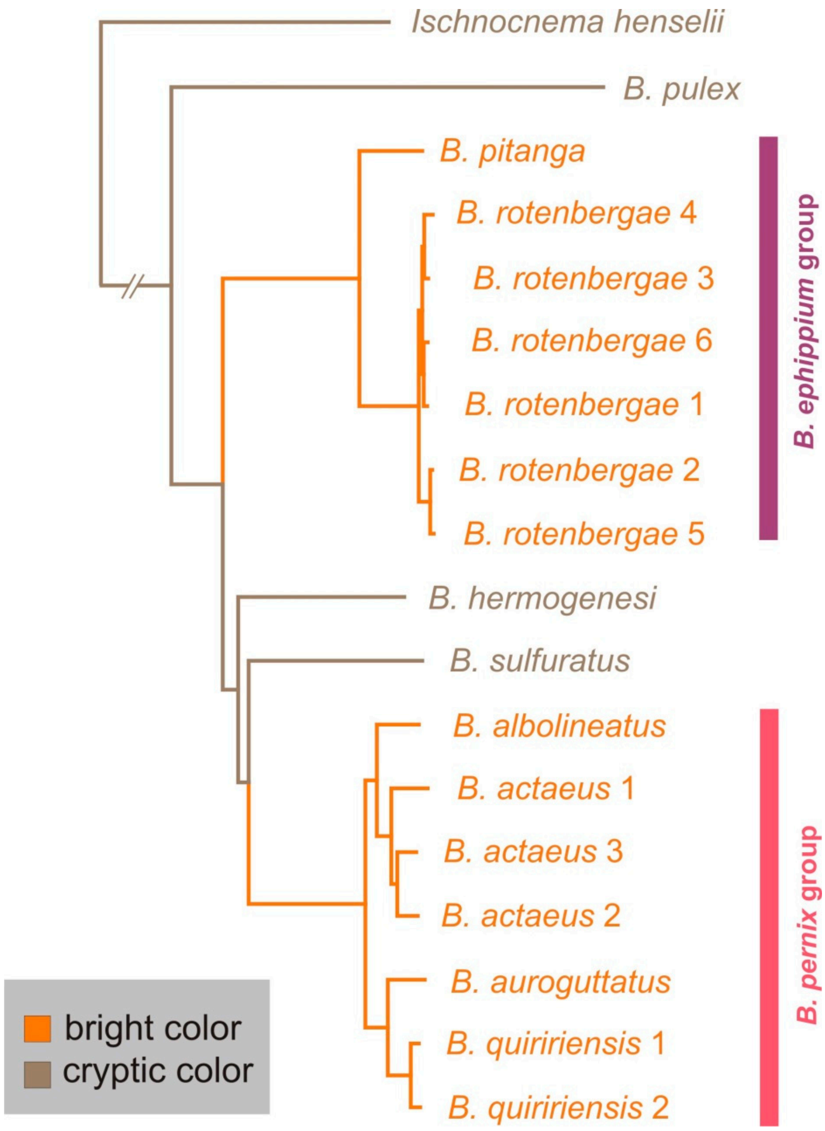

Figure S4. *Brachycephalus* phenotype reconstructed under a parsimony approach using TNT (Fitch optimization). Identical reconstructions were obtained for color (shown), and for bufoniform vs. leptodactyliform morphology. The character state “bright color” defines species that have at least some part of the body surface colored bright orange (dorsum, hands and feet, or ventral surface).

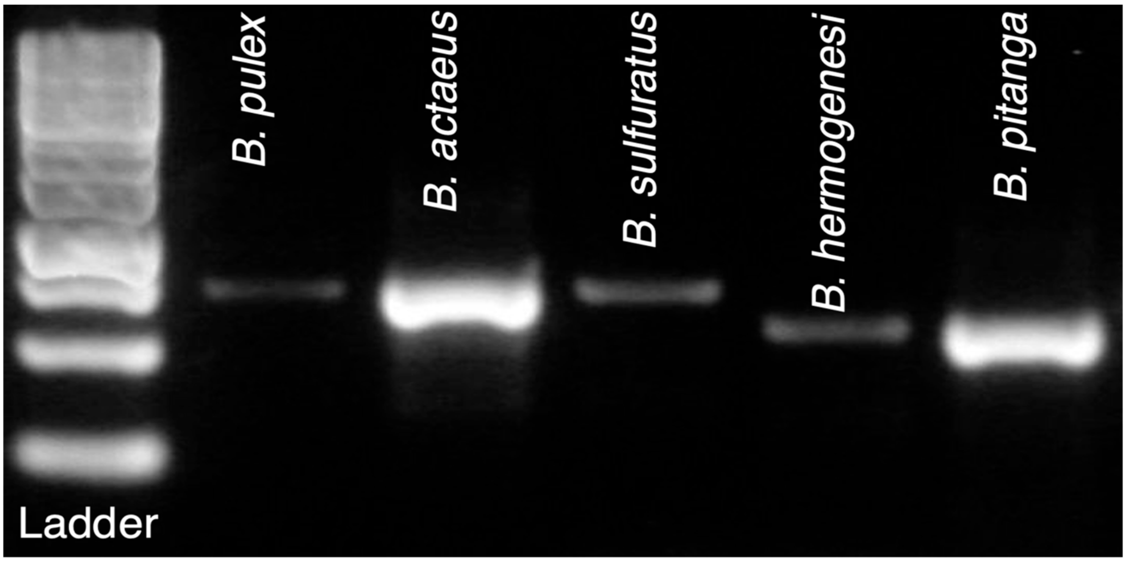

Figure S5. Electrophoresis 1.5% agarose gel showing different length fragment for the tRNA WANCY cluster region of different *Brachycephalus* species. Both *B. hermogenesi* and *B. pitanga* [resented smaller fragments as expected based on the tRNAs translocations.

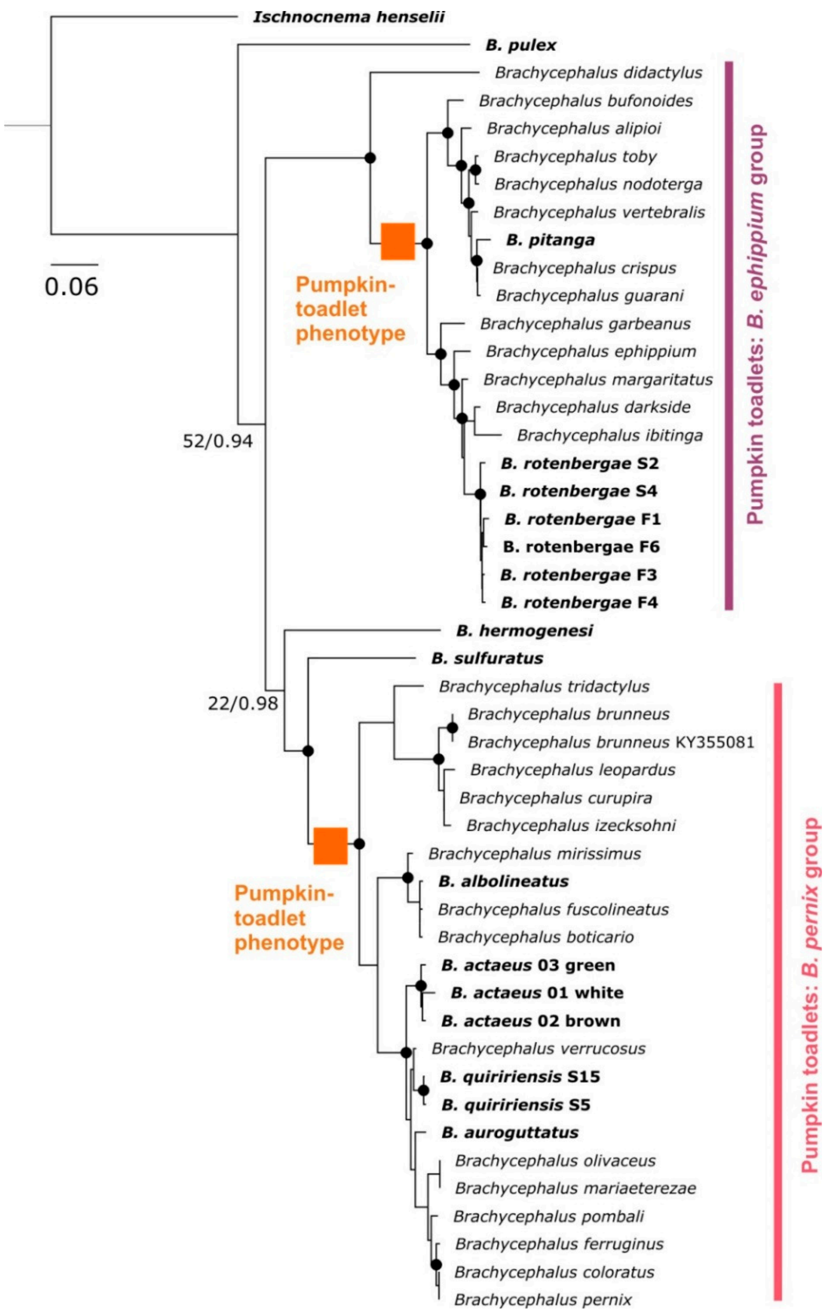

Figure S6. Maximum Likelihood phylogenetic inference for *Brachycephalus* species including mitogenomes obtained here and sequence data from GenBank (see Table S3 for additional accession numbers). Maximum likelihood non-parametric bootstrap support (BS) and posterior probabilities (PP) are shown near the branches for deep nodes (to the left and to the right of the bar, respectively). Black dots indicate fully supported clades (BS=100%, PP=1.0) and support values are not shown for nodes with low support (BS <0.9; PP<0.95) within each pumpkin-toadlet clade are not shown. Taxa in bold are those with complete mitogenomes obtained in the present study. Colored squares mark the two clades of pumpkin toadlet phenotype but do not symbolize evolutionary transitions.**Table S1.** List of samples used in the present analysis, their geographic provenance, sample/voucher number, body plan characteristic, mitogenome accession number and amont of reads analysed. Note that for most specimens, the full sample was used for DNA/RNA extraction and no separate voucher specimen was preserved. mtDNA reads: total number of reads recovered with MITObim; Total reads: Total of interleaved and non-duplicated RNAseq reads obtained for each specie.

Table S1. List of samples used in the present analysis, their geographic provenance, sample/voucher number, body plan characteristic, mitogenome accession number and amont of reads analysed. Note that for most specimens, the full sample was used for DNA/RNA extraction and no separate voucher specimen was preserved. mtDNA reads: total number of reads recovered with MITObim; Total reads: Total of interleaved and non-duplicated RNAseq reads obtained for each specie.

Species	Locality	Sample ID	Latitude	Longitude	Name in trees	Body plan	mtDNA acc. No.	mtDNA reads	Total reads
Ischnocnema henselii	Morro Boa Vista, Jaraguá do Sul, SC	Feb2017-RNA-A-FeedExp-C	-26.51611°	-49.05389°	*I. henselii*	--	MZ770752	426708	25247956
Brachycephalus pulex	PN Serra das Lontras, Arataca, BA	MVTIS 3204	-15.16198	-39.34381	*B. pulex*	leptodactyliform	MZ770742	266248	17702335
Brachycephalus hermogenesi	PESM Santa Virginia, São Luiz do Paraitinga, SP	MVTIS 3210	-23.33867°	-45.14815°	*B. hermogenesi*	leptodactyliform	MZ770740	618797	38589735
Brachycephalus sulfuratus	Casarão Backmeyer, São Francisco do Sul, SC	Feb2017-RNA44	-26.17528°	-48.71222°	*B. sulfuratus*	leptodactyliform	MZ770751	322432	12120096

Copyright: © 2021 by the authors. Licensee MDPI, Basel, Switzerland. This article is an open access article distributed under the terms and conditions of the Creative Commons Attribution (CC BY) license (https://creativecommons.org/licenses/by/4.0/).

Brachycephalus actaeus	Morro do Cantagalo, São Francisco do Sul, SC	MVTIS 3206 white strip	-26.18928°	-48.73229°	*B. actaeus* 1	bufoniform	MZ770735	447693	46194049
Brachycephalus actaeus	Estrada do Saí, São Francisco do Sul, SC	Feb2017-RNA56-green	-26.20167°	-48.69361°	*B. actaeus* 2	bufoniform	MZ770737	341395	17444017
Brachycephalus actaeus	Casarão Backmeyer, São Francisco do Sul, SC	Feb2017-RNA58-orange/brown	-26.17528°	-48.71222	*B. actaeus* 3	bufoniform	MZ770736	315185	14296301
Brachycephalus albolineatus	Morro Boa Vista, Jaraguá do Sul, SC	Feb2017-RNA02	-26.51611°	-49.05389°	*B. albolineatus*	bufoniform	MZ770738	306481	13310687
Brachycephalus auroguttatus	Pedra da Tartaruga, Garuva, SC	MVTIS 3207	-26.00583°	-48.92361°	*B. auroguttatus*	bufoniform	MZ770739	395248	31667155
Brachycephalus quiririensis	Campos do Quiriri, Garuva, SC	Feb2017-RNA-D FeedExp	-26.02556°	-48.97333°	*B. quiririensis* 1	bufoniform	MZ770743	377825	30154214
Brachycephalus quiririensis	Campos do Quiriri, Garuva, SC	Feb2017-RNA-C FeedExp	-26.02556°	-48.97333°	*B. quiririensis* 2	bufoniform	MZ770744	221775	18034035
Brachycephalus pitanga	PESM Santa Virginia, São Luiz do Paraitinga, SP	MVTIS 3205	-23.33°	-45.14°	*B. pitanga*	bufoniform	MZ770741	1132042	38736692
Brachycephalus rotenbergae	Mogi das Cruzes, SP, Brazil	MVTIS 3213	-23.49°	-46.19°	*B. rotenbergae* 1	bufoniform	MZ770745	1244504	24982826
Brachycephalus rotenbergae	Mogi das Cruzes, SP, Brazil	Brachy2 Ftcontrol	-23.49°	-46.19°	*B. rotenbergae* 2	bufoniform	MZ770749	711077	15084723
Brachycephalus rotenbergae	Mogi das Cruzes, SP, Brazil	MVTIS 3214	-23.49°	-46.19°	*B. rotenbergae* 3	bufoniform	MZ770746	1141170	21059190
Brachycephalus rotenbergae	Mogi das Cruzes, SP, Brazil	MVTIS 3215	-23.49°	-46.19°	*B. rotenbergae* 4	bufoniform	MZ770747	818811	33299329

Brachycephalus rotenbergae	Mogi das Cruzes, SP, Brazil	Brachy3 FT-infected	-23.49°	-46.19°	*B. rotenbergae* 5	bufoniform	MZ770750	904389	13876164
Brachycephalus rotenbergae	Mogi das Cruzes, SP, Brazil	MVTIS 3216	-23.49°	-46.19°	*B. rotenbergae* 6	bufoniform	MZ770748	1111026	21930389

Table S2. Best partition scheme selected with PartitionFinder2 for mitogenome tree analyses.

Subset	Best Model	# sites	Partition
1	GTR+G	977	12S
2	GTR+G	1664	16S
3	GTR+I+G	1127	COIII_codon_pos1, ND4_codon_pos1, ND1_codon_pos1, ND4L_codon_pos1
4	GTR+G	1627	COIII_codon_pos2, ND5_codon_pos2, ND4_codon_pos2, ND1_codon_pos2
5	GTR+I+G	864	ND1_codon_pos3, ND4_codon_pos3, ND4L_codon_pos3
6	GTR+G	575	ATP6_codon_pos1, ND2_codon_pos1
7	GTR+G	1171	CytB_codon_pos2, ND4L_codon_pos2, ND2_codon_pos2, ATP6_codon_pos2, ND3_codon_pos2
8	GTR+I+G	399	ND2_codon_pos3, ATP8_codon_pos3
9	GTR+G	513	COI_codon_pos1
10	GTR+I+G	513	COI_codon_pos2
11	GTR+I+G	512	COI_codon_pos3
12	GTR+I+G	781	COII_codon_pos1, CytB_codon_pos1, ND3_codon_pos1, ATP8_codon_pos2
13	GTR+G	229	COII_codon_pos2
14	GTR+I+G	829	COII_codon_pos3, ND5_codon_pos3
15	GTR+I+G	55	ATP8_codon_pos1
16	GTR+I+G	345	ATP6_codon_pos3, ND3_codon_pos3
17	GTR+I+G	643	COIII_codon_pos3, CytB_codon_pos3
18	GTR+G	601	ND5_codon_pos1
19	GTR+G	171	ND6_codon_pos1
20	GTR+G	171	ND6_codon_pos2
21	GTR+I+G	171	ND6_codon_pos3

Table S3. GenBank accession number for sequences included in the *Brachycephalus* spp. tree analyses. * mitogenome available: for this specimen we also included all other coding genes in the Maximum likelihood phylogenetic inference analyses.

Terminal name	Body plan	Voucher	12S rRNA	16S rRNA	CytB
Brachycephalus didactylus	leptodactyliform	MNRJ68818	MK697418.1	MK697363.1	MK697465.1
Brachycephalus alipioi	bufoniform	CFBH11759	MK697414.1	MK697360.1	MK697462.1
Brachycephalus boticario	bufoniform	Bboticario1		KX025370.1	
Brachycephalus brunneus	bufoniform		HQ435677.1	HQ435691.1	HQ435704.1
*Brachycephalus brunneus**	bufoniform	mitogenome	KY355081	KY355081	KY355081
Brachycephalus bufonoides	bufoniform	ZUFRJ15424		MH259790	
Brachycephalus coloratus	bufoniform	ind1		MT928335.1	
Brachycephalus crispus	bufoniform	CFBH27859	MK697415.1	MK697361.1	MK697463.1
Brachycephalus curupira	bufoniform	BSalto3		KX025380.1	
Brachycephalus darkside	bufoniform	PPGT600		MT734440.1	
Brachycephalus ephippium	bufoniform	CFBH12355	MK697421.1	MK697367.1	MK697467.1
Brachycephalus ferruginus	bufoniform		HQ435681.1	HQ435695.1	HQ435708.1
Brachycephalus fuscolineatus	bufoniform	Bfuscolineatus5		KX025340.1	
Brachycephalus garbeanus	bufoniform	CFBH12688	MK697424.1	MK697370.1	MK697470.1
Brachycephalus guarani	bufoniform	CFBH12997	MK697425.1	MK697371.1	MK697471.1
Brachycephalus ibitinga	bufoniform	CFBH44449		MT734431.1	
Brachycephalus izecksohni	bufoniform		HQ435683.1	HQ435696.1	HQ435710.1
Brachycephalus leopardus	bufoniform	Bleopardus1		KX025239.1	
Brachycephalus margaritatus	bufoniform	CFBH2420	MK697427.1	MK697377.1	MK697475.1
Brachycephalus mariaterezae	bufoniform	Bmariaterezae1		KX025316.1	
Brachycephalus mirissimus	bufoniform			MH136570.1	
Brachycephalus nodoterga	bufoniform	CFBH34912	MK697429.1	MK697379.1	MK697477.1
Brachycephalus olivaceus	bufoniform	CFBH40440	MK697432	MG889435.1	
Brachycephalus pernix	bufoniform		HQ435685.1	HQ435698.1	HQ435712.1
Brachycephalus pombali	bufoniform		HQ435687.1	HQ435700.1	HQ435714.1
Brachycephalus toby	bufoniform	CFBH44397	MK697436.1	MK697387.1	MK697484.1
Brachycephalus tridactylus	bufoniform	TRID-1		MG889431.1	
Brachycephalus verrucosus	bufoniform	Bverrucosus9		KX025234.1	
Brachycephalus vertebralis	bufoniform	CFBH1389	MK697442.1	MK697393.1	MK697490.1
